# Stratégie transfusionnelle des hémorragies graves du post-partum: étude rétrospective à propos de 47 cas

**DOI:** 10.11604/pamj.2016.25.169.7095

**Published:** 2016-11-16

**Authors:** Hosni Khouadja, Wissem Rouissi, Mohamed Mahjoub, Jaballah Sakhri, Dhafer Beletaifa, Khaled Ben Jazia

**Affiliations:** 1 Service Anesthésie Réanimation chirurgicale, Centre Hospitalo-Universitaire Farhat Hached Sousse-Tunisie; 2 Unité de Recherche UR12SP32, CHU Farhat Hached, Sousse, Tunisie; 3 Service d’Hygiène Hospitalière. Centre Hospitalo-Universitaire Farhat Hached Sousse, Tunisie

**Keywords:** Hémorragie grave du Post Partum, Transfusion sanguine, Morbi-mortalité maternelle, Severe postpartum hemorrhage, blood transfusion, maternal morbidity and mortality

## Abstract

**Introduction:**

L’hémorragie du post-partum est la principale cause de morbi-mortalité maternelle dans le monde. La prise en charge est multidisciplinaire. La stratégie transfusionnelle est capitale jouant un rôle majeur dans le pronostic maternel. L’objectif de ce travail a été de déterminer le rapport PFC/CGR lors de la prise en charge des hémorragies graves du post-partum.

**Méthodes:**

Une étude Etude rétrospective sur une période de 4 ans (2009-2012) a été réalisée dans un centre de maternité de référence de niveau III du centre-Est tunisien. Elle a inclut les parturientes admises pour une hémorragie sévère du post-partum définit par la nécessité d’une transfusion de plus de 04 CGR durant les 3 premières heures ou de plus de 10 CGR durant les 24 premières heures de prise en charge.

**Résultats:**

Notre étude a inclut 47 parturientes. Le diagnostic de l’HPP a été fait devant un saignement vaginal dans 28 cas et suite à une césarienne dans 19 cas. En préopératoire le taux d’Hb a été de 6.3 g/dl. Le rapport transfusionnel (PFC/CGR) a été de 1/0.7.

**Conclusion:**

Au cours de notre prise en charge, le rapport transfusionnel a été plus élevé que les recommandations récentes de la littérature stipulant une administration précoce et massive de PFC avec un ratio PFC/CGR compris entre 1/2 et 1/1. L’administration du fibrinogène (Fbg) et de l’acide tranexamique doit être précoce. L’emploi du facteur VII activé recombinant (rFVIIa) doit rester une solution ultime de prise en charge.

## Abstract (en)

**Introduction:**

Postpartum haemorrhage is the leading cause of maternal morbidity and mortality worldwide. It requires a multidisciplinary approach. Transfusion strategy is essential, playing a key role in maternal prognosis. This study aims to determine FFP/RBC ratio (plasma frais congelé/concentrés de globules rouges; fresh frozen plasma/red blood cells) during the treatment of serious postpartum haemorrhages.

**Methods:**

We conducted a retrospective study at a Maternity Referral Center (level III) in eastern Tunisia over a period of 4 years (2009-2012). All parturients admitted due to severe postpartum bleeding requiring transfusion of more than 4 Units of RBC during the first 3 hours or of more than 10 Units of RBC during the first 24 hours of treatment were included in the study.

**Results:**

47 parturients were enrolled in our study. The diagnosis of PPH was made on the basis of vaginal bleeding in 28 cases and following cesarean section in 19 cases. Preoperative hemoglobin level was of 6.3 g/dl. Transfusion ratio (FFP/RBC) was 1/0.7.

**Conclusion:**

During tratment transfusion ratio was greater than that indicated in the existing guidelines stating an early and massive administration of FFP with a FFP/RBC ratio ranging between 1/2 and 1/1. Fibrinogen (Fbg) and tranexamic acid should be administered as early as possible. The use of recombinant activated factor VII (rFVIIa) should remain the ultimate treatment option.

## Introduction

L’hémorragie du post partum (HPP) est classiquement définie par une perte sanguine supérieure à 500 millilitres (ml) en cas d’accouchement par voie basse et supérieure à 1000 ml en cas d’accouchement par césarienne [1]. La société canadienne des gynéco-obstétriciens (SOGC) considère l’HPP comme étant toute perte sanguine excessive survenant dans les 24 premières heures après la délivrance et susceptible d’entrainer une instabilité hémodynamique. Elle est sévère si la perte sanguine est supérieure à 1000 ml durant les premières 24 heures [2]. Elle peut survenir de façon inopinée chez toute parturiente sans qu’aucuns signes ou symptômes prédictifs ne soient présents. Les principales étiologies sont l’atonie utérine, la rétention placentaire et le traumatisme du tractus génital. C’est la principale cause de morbi-mortalité maternelle dans le monde [3]. Sa fréquence est en augmentation aussi bien dans les pays développés tel que le Canada, les Etats unis, l’Australie que dans les pays en voie de développement [1]. En France, sa fréquence est estimée à 5% toutes délivrances confondues [4]. Selon l’organisation Mondiale de la santé (OMS), elle est la cause du quart des décès maternelle dans le monde et à l’origine de 125 000 décès par an soit un décès maternel toutes les 4 minutes [5].

La prise en charge de l’HPP est multidisciplinaire. Elle est à la fois obstétricale, médicale, chirurgicale et fait intervenir la radiologie interventionnelle. Dans cette stratégie thérapeutique, la transfusion sanguine occupe une place prépondérante jouant un rôle capital dans le pronostic maternel. En effet, l’HPP est souvent accompagné d’une coagulation intra vasculaire disséminée de dilution et/ou de consommation dans un contexte d’acidose et d’hypothermie compliquant les conditions d’hémostase. De plus, il est actuellement établi qu’au moment du diagnostic, un taux plasmatique de fibrinogène inférieur à une valeur de 2g/l est prédictif de la sévérité du saignement [4]. En situation clinique, nous sommes souvent confrontés au dilemme du moment et du nombre de culots globulaires (CGR), de plasma frais congelé (PFC), de culots plaquettaires (CP) et des autres facteurs de coagulations qu’il faut administrer. Les recommandations actuelles de la prise en charge transfusionnelle des hémorragies graves du post-partum préconisent l’apport précoce et intensif de plasma avec un ratio PFC/CGR compris entre 1/2 et 1/1. L’adjonction du fibrinogène (Fbg) et de l’acide tranexamique doit être précoce. L’emploi du facteur VII activé recombinant (rFVIIa) doit rester une solution ultime de prise en charge [3]. L’objectif de ce travail a été de déterminer le rapport transfusionnel PFC/CGR/CP au cours de notre stratégie de prise en charge des HPP graves dans un centre de maternité hospitalo-universitaire de référence du centre Est de la Tunisie de niveau III.

## Méthodes

Une étude d’observation descriptive et rétrospective a été menée dans un centre de maternité de niveau III d’un établissement hospitalo universitaire de santé publique du Centre Est de la Tunisie (Centre Hospitalo-Universitaire Farhat Hached de Sousse Tunisie). Elle a concerné les parturientes admises pour une hémorragie de la délivrance et prise en charge conjointement par les services d’anesthésie-réanimation chirurgicale, de gynéco-obstétrique et par la banque du sang au cours d’une période de 4 ans allant de janvier 2009 à décembre 2012. L’admission de toutes parturientes en réanimation pour prise en charge d’une HPP sévère, a été, le principal critère d’inclusion de l’étude. La sévérité de l’HPP a été définie par la nécessité d’une transfusion massive: 04 culots globulaires ou plus au cours des trois premières heures du diagnostic ou 10 culots globulaires ou plus au cours des 24 premières heures de la prise en charge [6, 7]. L’étude s’est déroulée sur deux étapes: nous avons recensé les parturientes admises dans le centre de maternité pour accouchement et en réanimation pour complément de prise en charge d’une HPP, puis nous avons consulté la base des données informatiques de la banque du sang et identifié les parturientes ayant nécessité une transfusion massive. Après avoir fait le croisement des résultats, nous avons consulté et procédé à l’analyse des dossiers médicaux des patientes. Ainsi, nous avons pu notifier les données démographiques des parturientes (âge, indice de masse corporelle (IMC), groupe sanguin), les antécédents médico-chirurgicaux et obstétricaux (parité, terme, nombre de fœtus), le lieu et le mode d’accouchement (voie basse ou voie haute (programmée ou urgence)) et l’étiologie du saignement (atonie utérine, rétention placentaire, traumatisme de la filière génitale, une anomalie de l’insertion placentaire). Les paramètres cliniques (fréquence cardiaque, pression artérielle systolique et diastolique, la présence de signes de choc hypo volumique (marbrures des genoux, froideur des extrémités, temps de recoloration (TRC) > 3seconde)), la diurèse horaire, la température corporelle (°C) et l’estimation de l’importance du saignement, ainsi, que la détermination des données biologiques (taux d’hémoglobine (g/dl), taux de plaquettes (éléments/mm3), bilan d’hémostase (Taux de Prothrombine, Temps de Céphaline Activé, Taux de fibrinogène (g/l), calcémie (mmol/l) et fonction rénale) ont été également recueillis. Les modalités de la prise en charge médico-chirurgicale ainsi que la stratégie de prise en charge transfusionnelle avec le nombre de culots de globules rouges (CGR), de plasma frais congelé (PFC), de plaquettes (CP) administrés au cours des premières 24 heures de prises en charge ainsi que la nécessité d’administration des autres produits de la coagulation (fibrinogène, acide tranexamique, facteur VII activé recombinant) ont été rapportées. Enfin, nous avons calculé le rapport transfusionnelle PFC/CGR/CP sur la base qu’une unité de PFC transfusé est l’unité de référence [1]. La saisie et l’analyse des données ont été faites à l’aide du logiciel EpiInfo 6.0.

## Résultats

**Figure 1:**
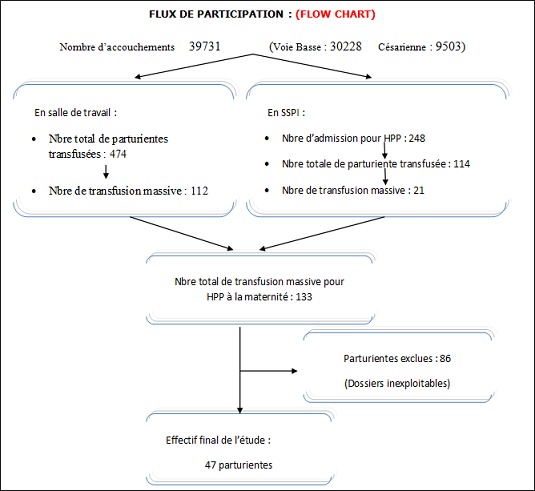
Flux de participation

**Figure 2:**
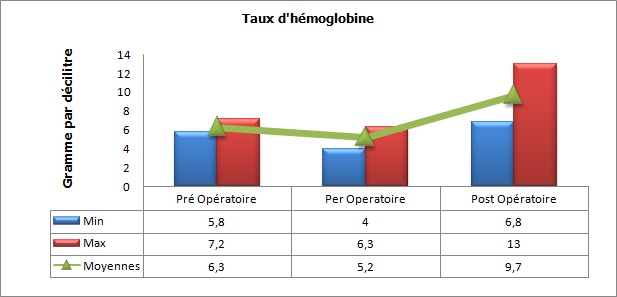
Evolution du taux d’hémoglobine (g/dl) des parturientes en péri-opératoire

**Figure 3:**
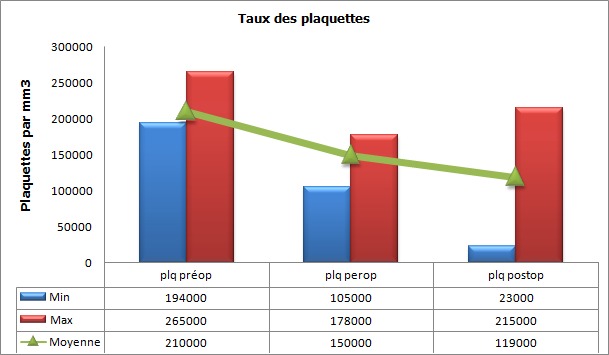
Evolution du taux des plaquettes (éléments/mm3) des parturientes en péri-opératoire

**Figure 4:**
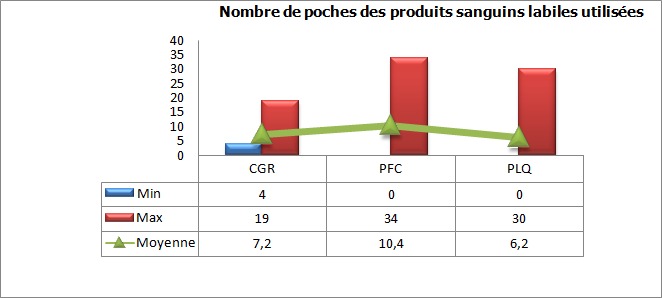
Nombre de poches des produits sanguins labiles administrées aux parturientes en péri-opératoire

**Figure 5:**
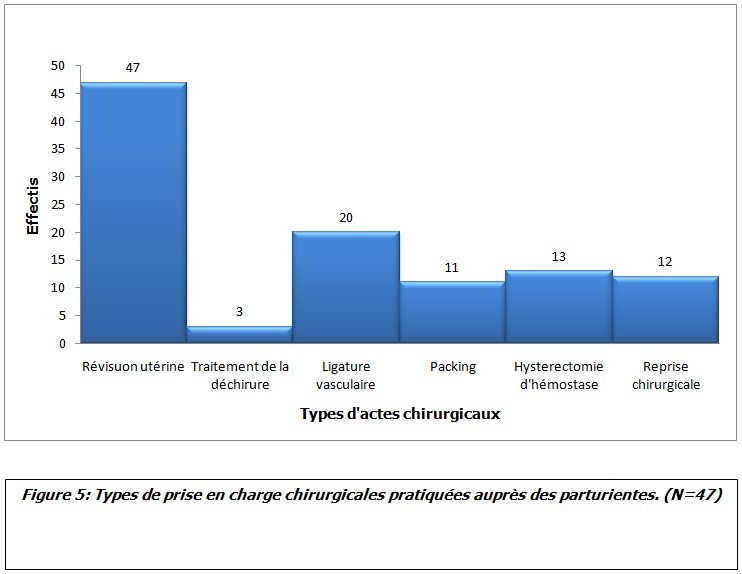
Types de prises en charge chirurgicales pratiquées auprès des parturientes (N=47)

Au cours de la période d’étude, notre série a inclut 47 cas d’hémorragies sévères du post-partum (Figure 1). L’âge moyen des parturientes a été de 31.56 années (avec des extrêmes de 18 et 44 ans), l’indice de masse corporel (IMC) moyen a été de 30.4 (avec des extrêmes de 23 à 35). L’âge gestationnel moyen en semaine d’aménorrhée (SA) a été de 39 SA (avec des extrêmes de 32 à 40). Les données médico-chirurgico-obstétricales des parturientes sont rapportées dans le Tableau 1. Le diagnostic de l’hémorragie de la délivrance a été porté devant un saignement jugé pathologique au cours d’une césarienne dans 40,42% des cas (19 parturientes) et suite à un accouchement par voie basse dans 59,57% des cas (28 parturientes). Aucune parturiente n’a bénéficié d’une estimation quantitative de l’importance du saignement. L’HPP a été rattachée à une atonie utérine dans 57,4% cas (27 parturientes), une rétention placentaire dans 27,6% cas (13 parturientes) et à une anomalie d’insertion placentaire à type de placenta prævia dans 8% cas (04 parturientes). Un traumatisme de la filière vaginale suite à un accouchement par voie basse avec extraction instrumentale par forceps a été noté dans 7% cas (03 parturientes). Au moment du diagnostic, le taux moyen de l’hémoglobine a été de 6.3 g/dl aves des extrêmes de 5.8 et 7.2 g/dl, et celui des plaquettes de 210000 éléments/mm^3^ avec des extrêmes de 194000 et 264000/mm^3^ (Figure 2, Figure 3). Seul 4 parturientes (8% des cas) ont bénéficié d’un dosage du taux de fibrinogène en préopératoire. Le taux moyen a été de 3.5g/l avec des extrêmes de 3.3 et 3.9g/l (Tableau 2). Toutes nos parturientes ont eu une dose maximale d’ocytocine (45 UI en IntraVeineux Lent (IVL)), au moins 500 µg de sulprostone et une révision utérine systématique sous anesthésie générale. Elles ont toutes été transfusées par des CGR iso-groupe iso-rhésus. Le nombre moyen de CGR transfusé a été de 7,2 CGR avec des extrêmes de 4 et 19 culots. Le nombre moyen de culots de PFC a été de 10,4 avec des extrêmes de 0 et 34 culots. Le nombre moyen de culots plaquettaires variait entre 0 et 30 culots soit une moyenne de 6,2 (Figure 4). Si l’on considère le plasma frais congelé comme étant une unité de référence, le rapport transfusionnel PFC/CGR/CP a été de 1/0,7/0,6 dans les 24 premières heures qui ont suivie le diagnostic. Une dose moyenne de 2 grammes de fibrinogène et d’acide tranexamique a été administrée à 17 et 9 parturientes respectivement (doses extrêmes de 1 et 4 grammes). Le recours au facteur VII activé recombinant a été nécessaire pour seulement 4,25% des cas (02 parturientes). Une hystérectomie d’hémostase a été indiquée dans plus du quart des cas (27,65% des cas soit pour 13 parturientes) (Figure 5). Aucune parturiente n’a bénéficié d’un traitement par radiologie interventionnelle. Deux parturientes de notre population d’étude (4,25% des cas) ont eu une évolution fatale après des délais de réanimation, respectivement, de 3 et 5 jours (Tableau 3).

**Tableau 1:** Caractéristiques médico-chirurgico-obstétricales des parturientes (N=47)

Caractéristiques étudiées	Types		Pourcentage
Antécédents médicaux	HTA chronique		4,25
	DNID		2,12
Antécédents chirurgicaux	Cholécystéctomie		4,25
	Césarienne		21,27
	IVG		4,25
	GEU		2,12
Terme	A terme		55,31
	Prématurité		44,68
Parité	Primipare		57,44
	Multipare		42,55
Nombre de fœtus	Monofoetale		82,97
	Multiple		17,02
Déroulement de la grossesse	Normale		76,59
	Pathologique	RPM	10,63
		Placenta prævia	8,51
		Pré éclampsie	4,25
Lieux d'accouchement	Intra muros		82,97
	Transfert secondaire		17,02
Mode d'accouchement	Voie basse spontanée		42,55
	Voie basse + extraction instrumentale (forceps)		8,51
	Césarienne		48,93

**Tableau 2:** Spécificités du bilan biologique péri-opératoire des parturientes (N=47)

	Pré opératoire (Moyenne [étendu])	Per opératoire (Moyenne [étendu])	Post opératoire (Moyenne [étendu])
TP (%)	75 [47-100]	néant	70 [50-100]
TCA (sec.)	32 [30-34]	néant	33.7 [29-42.1]
Fibrinogène (g/l)	3.5 [3.3-3.9]	néant	3.7 [2.3-4.1]
Calcémie (mmole/l)	Néant	2.9 [1.8-4.2]	3.8 [2.4-5]
Créatininémie (µmol/l)	65 [40-157]	néant	72 [52-119]

**Tableau 3:** Evolution post opératoire des parturientes (N=47)

Evolutions	Types	Pourcentage
Favorable		80,85
Complications	Etat de choc septique	4,25
	Endométrite	2,12
	TRALI	4,25
	TACO	2,12
	Embolie pulmonaire	2,12
Décès		4,25

## Discussion

Au cours de notre travail le ratio PFC/CGR/CP a été de 1/0.7/0.6 témoignant d’une administration plus libérale de PFC au cours de notre stratégie de prise en charge des HPP. En effet, pour répondre à l’urgence hémorragique la transfusion sanguine reste la pierre angulaire de la stratégie de prise en charge des HPP. Elle permet d’adapter les soins obstétricaux et de réanimation à la surveillance étroite des fonctions vitales, du débit de saignement et de l’hémostase. Elle est indispensable non seulement pour remplacer la perte sanguine et maintenir l’oxygénation tissulaire, mais aussi pour corriger la coagulopathie de dilution et/ou de consommation qui compliquent fréquemment l’hémorragie obstétricale. Les données récentes de la littérature recommandent un apport précoce et massif des facteurs de la coagulation dans un ratio PFC/CGR compris entre 1/1 et 1/2 [3]. Ces recommandations ont été essentiellement établis suite aux récentes publications de traumatologie de guerre ou civile suggérant que l’emploi large et précoce de PFC lors des transfusions massives pourraient être associées à une meilleure survie et à une moindre morbidité des patients de traumatologie [8]. La première publication à suggérer un bénéfice du ratio paritaire est celle de Borgman en 2007 [9]. Les auteurs ont analysé de façon rétrospective les données d’un registre militaire de 246 blessés nécessitant une transfusion massive admis dans un hôpital militaire d’Iraq entre 2003 et 2005. Les patients transfusés avec un ratio élevé voisin de 1/1.4 avaient une mortalité hospitalière plus basse que les patients transfusés avec un ratio intermédiaire de 1/2.5 ou bas de 1/8 (mortalité respective de 19%, 34%, et 65%). Dans les suites de cette étude, les modalités transfusionnelles des blessés de guerre d’Iraq ont été l’objet d’une littérature abondante, mais toujours rétrospective tentant d’illustrer les avantages d’un apport massif et précoce avec un ratio PFC/CGR proche de 1 [10]. Toutefois, dans une revue générale de la littérature, Godier et ses collaborateurs ont nuancé les conclusions de ces études [10]. Pour ces auteurs, plusieurs biais méthodologique sont rattachés à ces études. Outre le procédé d’analyse rétrospective adopté par ces études, il existe un biais de survie car ce ratio est une variable qui évolue au cours du temps et si ce ratio est intégré dans les analyses statistiques comme une variable temps dépendante, le bénéfice de survie associé au ratio élevé n’est plus retrouvé: pour ces auteurs les non survivants ne sont pas décédés parce qu’ils ont eu un ratio bas mais plutôt ils ont eu un ratio bas parce qu’ils sont décédés précocement suite à leurs lésions graves. De plus, en traumatologie, l’analyse des grands registres civils de patient polytraumatisés donne des résultats plus mitigés sur le bénéfice éventuel d’une transfusion massive de PFC [11]. Dans le domaine de l’obstétrique, en particulier en cas d’hémorragie du post-partum, plusieurs auteurs reconnaissent un bénéfice à augmenter le ratio transfusionnel PFC/CGR [10]. Une hémorragie obstétricale massive, du fait de son caractère sévère, aigue et actif, peut induire une spoliation sanguine importante sans avoir immédiatement de conséquences sur les constantes biologiques. D’un autre côté, il est souvent critique, voire dangereux d’attendre le résultat des examens biologiques pour décider d’une transfusion sanguine. La coagulopathie très souvent associée dans le contexte obstétrical, peut rendre utile la prescription précoce de plasma frais congelé et de concentrés globulaires dans un rapport d’une unité pour une unité permettant de corriger rapidement l’anémie aigue, la coagulopathie et de limiter le remplissage vasculaire par cristalloïdes et colloïdes [12]. C’est le concept du « damage control ressuscitation » développé pour les patients polytraumatisés qui consiste en l’administration précoce de PFC à fin d’éviter la triade létale hypothermie-acidose-coagulopathie. Dans une analyse rétrospective de Pasquier et ses collaborateurs, portant sur 142 hémorragies du post-partum ayant nécessité une transfusion sanguine, les auteurs ont comparé deux groupes de parturientes selon que la prise en charge ait été médicale uniquement (ocytocine et sulprostone) et/ou invasive (chirurgicale et/ou radiologie interventionnelle) et ils ont fixé un seuil PFC/CGR à 1/2 pour définir un rapport transfusionnel élevé ou bas. Les auteurs ont noté que dans le groupe de parturientes traité médicalement uniquement, le rapport transfusionnelle PFC/CGR a été significativement plus élevé que dans le groupe ayant nécessité une prise en charge interventionnelle (1/1.2 versus 1/1.6 respectivement (p<0.05)). Ils ont conclut alors à l’existence d’un réel bénéfice à utiliser un rapport élevé (PFC/CGR) proche de 1/1, pour réduire les pertes sanguines, le recours à une stratégie invasive de prise en charge et pour améliorer la morbidité maternelle [3]. Pour Bonnet, plus que la détermination des quantités à transfuser, c’est la précocité de la transfusion en CGR, PFC et plaquettes qui pourrait avoir un réel impact sur la morbi-mortalité maternelle [12].

Au cours des HPP une diminution rapide et précoce du taux fibrinogène est observée [5]. Les principaux mécanismes de cette hypofibrinogénemie sont expliqués par la perte des facteurs de la coagulation au cours du saignement et par une coagulopathie de consommation suite à l’activation du processus de la coagulation. Accessoirement, une coagulopathie de dilution est incriminée à cause d’un remplissage vasculaire parfois excessif pour maintenir une volémie adéquate particulièrement lorsque des colloïdes sont utilisés induisant une anomalie de la polymérisation de la fibrine [10, 13, 14]. D’autre part, le caractère prédictif d’une diminution de la concentration du fibrinogène est un argument pour envisager un bénéfice à l’administration précoce de concentrés de fibrinogène au cours de la prise en charge des HPP. En effet, en fin de grossesse un état d’hypercoagulabilité physiologique se développe à fin de prévenir le saignement au cours d‘un accouchement. C’est ainsi que le taux moyen de fibrinogénémie chez une femme enceinte en fin de grossesse est d’environ 5g/l bien en dessus des taux normaux de 3g/l en dehors de la grossesse et que le seuil classiquement admis de 1g/l est remis en question [15, 16, 17]. Dans un travail prospectif multicentrique recherchant les paramètres biologiques prédictifs de la progression vers une HPP sévère, Charbit et son équipe, ont inclut 128 patientes déjà traitées par sulprostone, mais n’ayant pas encore reçu de produits sanguins. Après analyse multi variée, les auteurs ont démontré qu’une concentration de fibrinogène inférieure à 2 g/l au moment de la prise en charge est un facteur prédictif indépendant de gravité avec une valeur prédictive négative de 79% si le taux de fibrinogène >4 g/l et une valeur prédictive positive de 100% si le taux de fibrinogène ≤2 g/l [13]. Ces mêmes résultats ont été confirmés par une étude prospective multicentrique de Cortet, incluant 738 parturientes ayant eu une HPP après accouchement par voie basse. Les auteurs ont démontré qu’au moment de la prise en charge, un taux de fibrinogène entre 2-3 g/l multiplie par 2 le risque de survenue d’une hémorragie sévère du post-partum (OR =1.9) et par 12 si le taux de fibrinogène est inférieur à 2g/l ((OR=11.99) [17]. Selon Ducloy-Bouthors, maintenir un niveau de fibrinogène plus élevé pourrait être une option thérapeutique séduisante [15]. Dans ce contexte, l’administration de concentrés de fibrinogène pourrait corriger de façon rapide la concentration plasmatique du fibrinogène [15].

Actuellement, aucune étude ne permet de déterminer la posologie de concentrés de fibrinogène à administrer, ni le bénéfice à une administration précoce de fibrinogène au cours des HPP. En attendant les résultats de la FIB-TRIAL, seul étude prospective randomisée dont l’objectif est de rechercher le bénéfice d’une administration précoce de fibrinogène au cours des HPP [18], seul le seuil de concentration plasmatique au delà duquel il convient de corriger le taux plasmatique reste la cible à viser. Alors que les recommandations européennes suggèrent qu’une dose de 3 à 4 grammes de fibrinogène soit administrée pour un seuil inférieur à 2 g/l [13]; pour Cortet, un seuil de fibrinogène de 3 g/l parait être plus intéressant [17]. Dans notre étude, au moment du diagnostic, seule 4 parturientes ont bénéficié d’un dosage de fibrinogène. La valeur moyenne de fibrinogénémie a été de 3.5g/l. Néanmoins, au moment de la prise en charge, l’administration précoce de concentrés de fibrinogène a été réalisée chez 17 parturientes (36% cas) à une dose moyenne de 2g avec des extrêmes allant de 2 à 4g.

Les agents antifibrinolytiques ont prouvé leur efficacité pour réduire le saignement en cas de chirurgie cardio-vasculaire, orthopédique, hépatique et dans les traumatismes graves hémorragiques [19, 20, 21]. Ils ont récemment attiré une attention particulière dans le traitement de l’HPP [22]. L’étude multicentrique randomisé, EXADELI, réalisée en France, a démontré l’efficacité d’un bolus de 4 g d’acide tranexamique administré en IVL sur 1 heure suivie d’une perfusion d’entretien de 1 g/h sur 6 heures par rapport à un placebo au cours des accouchements par voie basse compliqués d’une hémorragie de la délivrance [22]. De même, la revue de la littérature de la Cochrane Data Base ^®^ en 2010 a confirmé l’effet préventif de l’acide tranexamique sur la survenue d’une HPP au cours des accouchements par voie basse et par césarienne [23]. Mais en attendant les résultats d’un large essai multicentrique international (World Maternal Antifibrinolitic) évaluant l’effet d’un traitement précoce par acide tranexamique sur la morbi-morbidité maternelle et le taux d’hystérectomie et en raison de son faible coût, l’OMS recommande que cette molécule soit utilisée au cours des HPP en cas d’échec des autres mesures à la dose de 1 g en IVL sur 1 à 5 minutes répétés une fois au bout de 30-60 minutes si le saignement persiste [22, 24].

L’utilisation du facteur VII activé recombinant (rFVIIa) a été largement décrite à travers des cas rapportés, de séries de cas ou de registres dans le contexte d’hémorragies sévères postopératoires ou post-traumatiques. Dans le traitement de l’HPP, l’expérience de l’utilisation du rFVIIa a été enrichie par les données de différents registres [25]. Les données recueillit témoignent de l’existence d’un réel bénéfice à utiliser le rFVIIa en cas de HPP sévère, lorsque l’indication est bien posée et que les effecteurs de l’hémostase sont à des taux convenables [26]. Ces résultats ont été suffisamment encourageants pour justifier les premières recommandations européennes sur son utilisation hors autorisation de mise sur le marché (AMM) dans l’hémorragie du Post-partum en 2006 et une autorisation temporaire thérapeutique par l’agence française de sécurité sanitaire des produits de santé (Afssaps) en 2007 [27]. En effet, le registre de l’Europe du Nord a colligé un effectif de 113 parturientes sur la période de 2000 à 2004 utilisant le rFVIIa dans l’HPP. Les auteurs ont noté l’efficacité de cette molécule dans 80% des cas lors d’une utilisation curative pour une administration, dans la majorité des cas unique, à la posologie moyenne de 90µg/Kg [26]. Une revue de la littérature dont trois registres (Européen, Italien et Australien) a inclut 272 cas de HPP sévère nécessitant le recours au rFVIIa hors AMM. Les auteurs ont trouvé qu’une dose unique de 81 µg/Kg était efficace dans 85% cas pour arrêter le saignement [28].

Cependant, le moment optimal d’utilisation du rFVIIa reste à éclaircir [29]. Geiger et al. suggèrent l’administration du rFVIIa en cas de saignement diffus et persistant malgré le recours aux utero toniques et à la transfusion massive [29]. Certains auteurs finlandais ont proposé son administration dès lors que le saignement dépassait 1,5 fois le volume sanguin maternel [30]. L’AFssaps recommande le recours au rFVIIa avant l’hystérectomie d’hémostase, en cas d’échec de l’embolisation ou avant celle-ci, lorsqu’elle est indiquée et non disponible immédiatement; enfin, en situation d’échec thérapeutique après chirurgie première voire même reprise chirurgicale [27]. La posologie recommandée est de 60-90 µg/kg en une prise unique [12, 28]. Une deuxième dose (90 à 120 µg/kg répétée 1 heure après la première dose) pourrait être utile si le saignement perdure en supplément des mesures d’hémostase invasives maximales (embolisation et/ou chirurgie) et si possible avant la réalisation d’une hystérectomie [28]. Enfin, si l’efficacité du facteur VII activé requiert la correction préalable de le numération plaquettaire (taux de plaquette> 50 G/l), de la concentration plasmatique du Fibrinogène (fibrinogène >2g), de l’acidose, de la calcémie et de la température ; il est fondamental de garder à l’esprit qu’il s’agit d’une « bombe à coaguler » et que le risque thromboembolique n’est pas nul et estimé à 1,4 à 2,5% des cas selon les séries [8, 12, 26]. Dans notre serie, nous avons eu recours au rFVIIa à seulement deux reprises, après une hystérectomie d’hémostase dans les deux cas. Un seul cas de décès est survenue suite à un état de choc septique et aucun accident thrombo-embolique n’a été noté.

Perspectives d’avenir: La récupération sanguine et le monitorage per-opératoire de l’hémostase représentent l’une des perspectives d’avenir de la prise en charge des hémorragies de la délivrance. Longtemps considérée comme contre-indiquée en obstétrique, la tolérance de la récupération sanguine per opératoire a été démontrée à travers des séries de cas clinique pour des césariennes à haut risque hémorragique [12, 15]. Ainsi la balance bénéfice-risque semble pencher actuellement en faveur de l’utilisation de cette technique en obstétrique, ce qui a conduit plusieurs sociétés savantes à émettre des recommandations favorables à l’utilisation de la récupération sanguine per opératoire en cas de HPP grave, et cela malgré l’absence d’essai randomisé contrôlé. Ducloy Bouthors et al., recommandent le cell-saver en cas d’anomalie d’insertion placentaire, d’utérus multi cicatriciel ou d’utérus sur distendu, d’hématome retro placentaire ou de mort in-utero prolongé [22]. Cependant des règles de sécurité entourant l’utilisation de cette technique doivent être respectées: utilisation de la canule d’aspiration en cas de césarienne uniquement après la délivrance complète du placenta, contre-indication en cas de plaie de la filière génitale ou périnéale, utilisation de filtres permettant une déplétion leucocytaire. Selon Bonnet et al., cette technique ne doit être utilisée que par une équipe entrainée [12]. Actuellement, en France, l’emploi du cell-saver reste limité aux cas d’hémorragie sans altératives transfusionnelles, c’est-à-dire en cas d’impossibilité de recevoir du sang homologue pour des raisons hématologiques, personnelles ou religieuses.

L’intérêt du monitorage per opératoire délocalisé de l’hémoglobine par Hémocue^®^ n’est plus à démontrer pour guider les besoins transfusionnels en CGR [31], par contre, celui de l’hémostase n’est pas encore de pratique courante. En effet, au cours des hémorragies graves du post partum, attendre les résultats du laboratoire pour prendre une décision thérapeutique fait courir à la femme enceinte un réel danger. D’une part, l’accès aux résultats des examens biologiques peut nécessiter au moins 45-60 minutes et d’autre part, une fois les résultats obtenus la situation hémorragique de la femme enceinte a déjà évolué. Or, durant ces « golden minutes » le monitorage de la coagulation sur sang total au bloc opératoire par Thromboélastométrie (ROTEM^®^, Tem international GmbH, Munich, Germany) ou Thromboélastographie (TEG^®^, haemaneticscorp, Braintree, MA, USA) peut guider le choix thérapeutique optimal pour la parturiente [8, 31, 32]. ROTEM^®^ et TEG^®^ permettent une étude globale et rapide de la cinétique de la coagulation depuis la formation du caillot jusqu’à sa dissolution. Les résultats sont disponibles au bout de 10 à 15 minutes [33]. Plusieurs études ont démontré une corrélation entre les résultats du laboratoire et celles du monitorage délocalisé de l’hémostase sur les différents paramètres [34]. Dans une récente étude multicentrique, De longe et ses collaborateurs ont démontré une bonne corrélation des paramètres du FIBTEM avec le taux de fibrinogène plasmatique sur un effectif de 161 parturientes confirmant les résultats des nombreux travaux rapportés précédemment [33].

La principale limite de notre étude est son caractère rétrospectif. De plus, en choisissant de définir la sévérité de l’hémorragie du post-partum par la nécessité d’une transfusion massive seulement, la taille de l’échantillon de la population d’étude ne nous a pas permis de dégager les facteurs pronostics de morbi-mortalité. Or, plusieurs critères définissant la sévérité de l’hémorragie de la délivrance existe dans la littérature. Il peut s’agir d’une chute du taux d’hémoglobine ≥4 g/dl, la nécessité d’une transfusion massive, le remplacement de plus de 50% de la masse sanguine en moins de 3 h, la nécessité d’une prise en charge interventionnelle chirurgicale (ligature vasculaire ou hystérectomie) et/ou radiologique (embolisation artérielle) ou bien le décès de la parturiente [17].

## Conclusion

La prise en charge des HPP est à la fois médicale, obstétricale, chirurgicale et radiologie interventionnelle. La stratégie transfusionnelle est capitale, jouant un rôle majeur dans le pronostic maternel. Un saignement parfois incoercible et des troubles de l’hémostase souvent précoce, à cause d’une coagulopathie de consommation et/ou de dilution, imposent un apport précoce et massif de facteurs de coagulation, de fibrinogène et d’agent antifibrinolytique. Le ratio PFC/CGR doit être paritaire. Le recours au facteur VII activé recombinant doit rester une solution ultime de prise en charge transfusionnelle.

### Etat des connaissances actuelles sur le sujet

- L’hémorragie du post partum est la première cause de morbi-mortalité maternelle dans le monde;
- Il est recommandé une prise en charge transfusionnelle précoce et intensive avec un ratio PFC/CGR compris entre 1/2 et 1/1.

### Contribution de notre étude à la connaissance

- Un apport précoce et plus libérale de produits sanguins labiles avec un rapport PFC/CGR > 1/1 serait plus bénéfique en cas d’hémorragie grave du post partum selon l’expérience de notre équipe;
- L’administration de fibrinogène et d’acide tranexamique doit être précoce.
